# Assessing the Association Between Genetic Variants in ACE, SOD1, and PER3 and their Role in Breast Cancer Risk among Jordanian Women

**DOI:** 10.7150/jca.133243

**Published:** 2026-06-10

**Authors:** Laith N. AL-Eitan, Fouad A. Almomani, Mohammed S. Alorjani, Maryam K. Alasmar, Haneen O. Ali, Mansour A. Alghamdi

**Affiliations:** 1 Department of Biotechnology and Genetic Engineering, Jordan University of Science and Technology, Irbid 22110, Jordan.; 2 Department of Pathology and Microbiology, College of Medicine, Jordan University of Science and Technology, Irbid 22110, Jordan.; 3 Department of Anatomy, College of Medicine, King Khalid University, Abha 62529, Saudi Arabia.; 4 Genomics and Personalized Medicine Unit, The Center for Medical and Health Research, King Khalid University, Abha 62529, Saudi Arabia.

**Keywords:** breast cancer, polymorphism, *ACE*, *SOD1*, *PER3*

## Abstract

**Background:**

Genetic and environmental factors regulate many physiological processes in the human body, and alterations in these processes may contribute to the development of various diseases, including breast cancer (BC), which is considered the most prevalent cancer among women and a leading cause of cancer-related mortality in the Jordanian population. Genes such as *ACE*, *SOD1* and *PER3* play important roles in regulating essential biological functions. These genes are involved in key physiological pathways, including blood pressure regulation, oxidative stress response and circadian rhythm maintenance, and genetic variants within them may influence susceptibility to cancer. Therefore, this study investigates the association between polymorphisms in the *ACE*, *SOD1* and *PER3* genes and the risk of breast cancer, with the aim of evaluating how these genetic variations relate to breast cancer susceptibility and clinical outcomes in Jordanian women.

**Methods:**

Blood samples of 300 women diagnosed with breast cancer, along with 300 healthy participants, were collected, and DNA was extracted from them. Genetic variants in the *ACE* (rs1799752), *SOD1* (rs36232792) and *PER3* (rs57875989) genes are examined through employing direct PCR to amplify the target regions.

**Results:**

The *ACE* (rs1799752) variant was observed to be associated with breast cancer susceptibility, with the I/I genotype increasing risk of breast cancer (OR = 5.138, 95% CI = 1.38-19.03, p = 0.014). No associations were observed for *SOD1* (rs36232792) and *PER3* (rs57875989) variants.

**Conclusion:**

The rs1799752 polymorphism is suggested to have the potential of serving as a biomarker for breast cancer susceptibility in Jordanian women, as it is associated with elevating the risk.

## Introduction

Breast cancer is the second leading cause of cancer-related deaths worldwide and is the most common cancer in women. It originates in breast cells, forming a tumor that can invade nearby tissues and metastasize to other parts of the body. Some alterations in breast cells can also lead to non-cancerous conditions, such as atypical hyperplasia, cysts, and benign tumors like intraductal papillomas 1. Breast cancer often starts in the ducts or lobules of the breasts, which contain milk-producing cells organized into lobes and connected to the nipple through ducts 2. Its development is influenced by genetic, hereditary, and various environmental risk factors. Treatment strategies depend on specific molecular features, including hormone receptor status (ER and PR), HER2 activation, gene mutations such as BRCA1/2 and PIK3CA, and immune markers such as TILs and PD-L1 3.

There are different molecular subtypes of breast cancer, such as Luminal A, Luminal B, HER2-enriched, and basal-like. These subtypes are based on mRNA gene expression profiles. Each subtype has its own biological traits that help in improving patient outcomes by employing a personalized approach in treatment 4. Several factors have been identified as significant risk factors for breast cancer, including genetic predisposition, family history, and reproductive factors such as age at menarche, parity and breastfeeding, as well as lifestyle-related factors including diet, obesity, smoking, alcohol consumption and exposure to radiation 5.

Angiotensin-converting enzyme (ACE) is an enzyme that controls several physiological processes in the cardiovascular system and is considered a dipeptidase enzyme, and its enzymatic activity is dependent on chloride and zinc ions, which are necessary for its action. While ACE exists in various forms, the primary isoform is a membrane-bound glycoprotein predominantly expressed in the lungs, anchored to the surfaces of cells that line blood vessels 6. To maintain fluid and electrolyte balance, ACE supports homeostasis through its role in the renin angiotensin system (RAS). The RAS operates via two primary pathways: ACE/Ang II/AT1R, which promotes vascular constriction and cell proliferation and ACE2/Ang1-7/MasR, which generally has opposing effects. Dysregulation of the renin-angiotensin system (RAS), particularly the ACE/Ang II/AT1R pathway, has been linked to several cancers, including breast, ovarian and prostate cancers, and contributes to key processes such as cell proliferation, angiogenesis and metastasis 7, 8.

SOD1 (Cu/Zn superoxide dismutase) is an enzyme that breaks down superoxide radicals (O₂⁻) produced in the mitochondria and cytosol, helping to control oxidative stress in cells. What distinguishes SOD1 from other human superoxide dismutases is its high concentration in the cytosol. The other two forms are Mn superoxide dismutase (SOD2), located exclusively in the mitochondria and extracellular superoxide dismutase (SOD3), which also binds copper and zinc and is found in different cellular compartments, with SOD3 attached to the extracellular matrix. 9. In addition to its well-established role as a superoxide dismutase, the SOD1 enzyme also exhibits peroxidase activity and possesses the capacity to bind DNA, suggesting additional roles in cellular processes 10. The protein features an eight-stranded β-barrel structure with a catalytic copper ion, a structural zinc ion, an electrostatic loop and an intramolecular disulfide bond between cysteines 57 and 146. Variants of this gene specifically caused by missense mutations could affect the protein structure and function and are of significant interest for research studies of the role of SOD1 in different diseases 11.

PER3 is a major component of the circadian clock that works with proteins like PERIOD and CRYPTOCHROME (CRY) to regulate the body's internal rhythm by inhibiting core clock gene expression 12. PER3 and PER1, PER2, CRY1, and CRY2 form complexes that move into the nucleus to suppress BMAL1 and CLOCK transcription factors. Disturbances in circadian rhythms are linked to higher cancer risk, with altered circadian gene expression being associated with cancer progression 13. *PER3* is in the 1p36 chromosomal region, frequently deleted in human cancers, particularly breast cancer 14, 15. Its expression fluctuates in peripheral tissues and organs as a component of the negative branch of the core molecular circadian clock feedback loop 16, 17, 18. Patients with breast and other cancers exhibit reduced PERIOD clock gene expression in their tumors compared to adjacent normal tissue 19, 20. This and additional data suggest that PERIOD clock genes function as tumor suppressors 14, 21.

The association between polymorphisms in the *ACE* (rs1799752), *SOD1* (rs36232792), and *PER3* (rs57875989) genes and breast cancer risk yielded varied results in different populations. Additionally, the research examining the combined impact of these specific genetic variants in breast cancer patients within the Jordanian population is insufficient. In this regard, this study seeks to address the gap by examining the prevalence of these genetic polymorphisms among Jordanian women. Moreover, this research also aims to explore the potential impacts of these genetic differences on breast cancer susceptibility as well as the survival rates of breast cancer patients.

## Methods

### Subjects

A case-control study was designed to explore the link between genetic polymorphisms in the *ACE*, *SOD1* and *PER3* genes and the risk of developing breast cancer. This study included 600 participants, with equal numbers of 300 breast cancer patients and 300 healthy controls who were not related to each other. Participants were recruited randomly from the chemotherapy clinics at King Abdullah University Hospital (KAUH) and King Hussein Medical Center. The study protocol was approved by the Human Ethics Committee of Jordan University of Science and Technology and KAUH (No.: 9/143/2021). Participant's informed consent was obtained prior to sample collection. Comprehensive data for the patients, including clinical history, demographic data, diagnostic results, and medication data, was collected from the electronic medical records of the patients. For the patients to be included in the study, they had to have a confirmed diagnosis of breast cancer based on histopathological results. In addition, the patients had to be negative for HIV, HBV, and HCV infections. Furthermore, the patients had to have comprehensive data recorded in the KAUH patient registry. Exclusion criteria included patients who failed to give consent for the study, patients who had undergone blood transfusions during surgery, and patients whose clinical documentation was inadequate.

### Candidate gene and polymorphisms selection

To select polymorphisms for this study, we focused on significant polymorphisms in the *ACE*, *SOD1* and *PER3* genes reported in breast cancer patients from diverse populations globally. The selected polymorphisms underwent further validation using various predictive online tools and specific web servers to assess their potential impact. These polymorphisms were compared and verified through several databases, including the NCBI SNP database (https://www.ncbi.nlm.nih.gov/snp/), HaploReg v4.2 (https://pubs.broadinstitute.org/mammals/haploreg/haploreg.php), Ensembl (http://www.ensembl.org/index.html), RegulomeDB (https://regulomedb.org/regulome-search/), rSNPBase 3.1 (http://rsnp3.psych.ac.cn/) and the SNP Function Prediction tool (https://snpinfo.niehs.nih.gov/snpinfo/snpfunc.html). This is to ensure that the polymorphisms used for the research were relevant and accurate.

The RegulomeDB database shows that the polymorphisms rs1799752 in the *ACE* gene, rs36232792 in *SOD1* and rs57875989 in *PER3* each received a score of 4, along with a rating of 0.60906. The results suggest that these polymorphisms could affect binding sites and contribute to the regulation of gene expression. Moreover, the HaploReg v4.2 database showed functional annotations of those genes, where the rs1799752 variant, located in the intronic region within the *ACE* gene, affects five different binding motifs. The rs36232792 in the *SOD1* gene is a synonymous coding SNV that potentially modifies twelve motifs, impacting histone marks in promoters and enhancers and influencing transcription factor binding sites. Meanwhile, the rs57875989 variant of the *PER3* gene alters the Zfp410 motif and could also influence histone marks in promoters and enhancers. These findings suggest that the polymorphisms in *ACE*, *SOD1* and *PER3* may indirectly contribute to breast cancer development by impacting the regulation of transcription factors associated with these genes.

### DNA extraction

Using the Puregene® Blood Core Kit A (Qiagen), genomic DNA was isolated from blood samples by adhering to the manufacturer's protocol. The isolated DNA purity and concentration were assessed with a NanoDrop spectrophotometer and verified through gel electrophoresis.

### Genotyping

Genotyping of the rs1799752 variant in the *ACE* gene, rs36232792 in *SOD1* and rs57875989 in *PER3* was conducted using direct PCR. Each PCR reaction was carried out in a total volume of 25 µL, which included nuclease-free water, a 2x master mix, 10 µM of both forward and reverse primers and template DNA. The specific primer sequences used for the current study have been previously reported 22-24. Further details on the reaction conditions can be found in Table 1. Genotyping quality was further validated by repeating the genotyping for randomly selected samples and verifying genotype consistency. Samples with unclear or ambiguous results were re-analyzed to confirm genotype assignment.

### Statistical analyses

Statistical analyses were conducted using various methods and tools to ensure reliable and accurate results. To assess Hardy-Weinberg equilibrium, allele and genotype frequencies, inheritance models and the relationship between haplotypes and disease status, the SNPstat tool (https://www.snpstats.net/start.htm) was utilized. In addition, odds ratios (OR) and 95% confidence intervals (CI) were determined, with statistical significance defined by p-values below 0.05. The Statistical Package for the Social Sciences (SPSS) software, version 26.0 (SPSS, Inc., Chicago, IL), was used to evaluate genotype-phenotype correlations using Pearson's chi-square test and one-way ANOVA. To account for rare genotypes with small sample counts, Fisher's exact test was applied when expected counts were less than five, while the chi-square test was used for comparisons with sufficient counts. Post hoc statistical power was estimated using G*Power (v3.1.9.7), utilizing the observed genotype frequencies in cases and controls, with a two-tailed test for comparisons between independent groups and an alpha level of 0.05. Multivariable binary logistic regression analysis was conducted to determine whether the studied polymorphisms were independent risk factors for disease susceptibility after adjustment for potential confounding variables. Survival analysis was performed using the Kaplan-Meier method in R (version 4.5.3), and differences between groups were assessed using the log-rank test. To adjust for multiple testing, the appropriate number of polymorphisms was calculated according to a method described in a prior study 25. The significance level was further refined using the Bonferroni correction, where it was set to α/n, with α = 0.05 and n representing the total number of tests performed 26.

## Results

### Patient characteristics

*ACE*, *SOD1*, and *PER3* gene polymorphisms were utilized in this case-control study to explore their association with the risk of developing breast cancer. 300 unrelated female patients with breast cancer and 300 unrelated healthy female controls from the Jordanian population were included in the study. The mean age of the breast cancer patients was 52.32 ± 11.39 years, with a median age of 51 years and an age range of 25-85 years. Moreover, 160 patients reported a family history of cancer, representing 56.34% of the breast cancer group.

### Hardy-Weinberg Equilibrium (HWE) and minor allele frequencies

The genetic variations rs1799752, rs36232792, and rs57875989 came under a thorough scrutiny of the minor allele frequencies in breast cancer patients and in normal persons, which was fundamental in defining the possible variations in the distribution of genetic variations between both categories, suggesting a possible link between them and the risk of breast cancer. The HWE test was applied to confirm the genetic information since it determines whether the data on the allele and genotype frequencies within a population fit expectations from random mating and is a fundamental marker of the genetic constitution of the investigated population.

For most of the polymorphisms analyzed, the genotype frequencies in both cases and controls were in consistent with Hardy-Weinberg equilibrium, indicating that the study populations were genetically stable and unlikely to be affected by factors such as selection, mutation, or genotyping errors that could alter allele distribution. However, the rs1799752 polymorphism in the *ACE* gene showed a clear deviation from Hardy-Weinberg equilibrium in both groups. Detailed data supporting these findings are presented in Table 2.

### Allele and genotype distributions and their association with breast cancer risk

Detailed information on allele frequencies and genotype distributions, along with unadjusted and adjusted odds ratios obtained using multivariable logistic regression analysis controlling for age, sex, BMI, and smoking status, is presented in Table 3. Associations of genotypes were evaluated under a codominant genetic model. For the *ACE* rs1799752 variant, the I/I genotype was associated with an increased risk of breast cancer in both unadjusted analysis (OR = 4.12, 95% CI: 1.34-12.62, p = 0.009) and after adjustment (adjusted OR = 5.14, 95% CI: 1.38-19.03, p = 0.014). While the *SOD1* rs36232792 variant, the D/D genotype, showed a significant association in the unadjusted analysis, this association was attenuated and no longer statistically significant after adjustment (adjusted OR = 7.21, 95% CI: 0.88-59.12, p = 0.066). No significant associations were observed for the *PER3* rs57875989 variant in either unadjusted or adjusted analyses.

### Analyses using different genetic models

The association between polymorphisms in the *ACE*, *SOD1*, and *PER3* genes and breast cancer risk was investigated through various genetic models. Odds ratios (ORs) for these associations are detailed in Table 4. Significant associations were observed for the *ACE* (rs1799752) polymorphism under the recessive inheritance models (OR = 3.82, P = 0.011). The post hoc power for this association was 0.78. In contrast, no significant association was found for the *SOD1* and *PER3* polymorphisms across the genetic models analyzed.

### The clinical features and genotypic associations of polymorphisms

As outlined in Table 5, statistical analyses were performed to explore the association between *ACE*, *SOD1*, and *PER3* polymorphisms and clinical outcomes in breast cancer patients. The results indicated that none of the *PER3* (rs57875989), *SOD1* (rs36232792), or *PER3* (rs57875989) polymorphisms were associated with the clinical trait of breast cancer (p > 0.0167).

### Logistic regression analysis

Multivariable binary logistic regression analysis was performed to evaluate whether the studied polymorphisms were independently associated with disease susceptibility after adjusting for potential confounding factors, including age, BMI, smoking status, age at menarche and family history of cancer. The results are presented in Supplementary Table S1.

For the *ACE* polymorphism, the I/I genotype was significantly associated with increased disease risk compared with the reference genotype (D/D) (OR = 5.14, 95% CI = 1.39-19.03, P = 0.014). In contrast, no significant associations were observed for genotypes of the *SOD1* or *PER3* polymorphisms (P > 0.05). However, family history of cancer was significantly associated with increased disease risk in all models (P < 0.001), while age, BMI, smoking status and age at menarche were not significant predictors.

### Survival analysis of breast cancer patients

**Figure 1:**
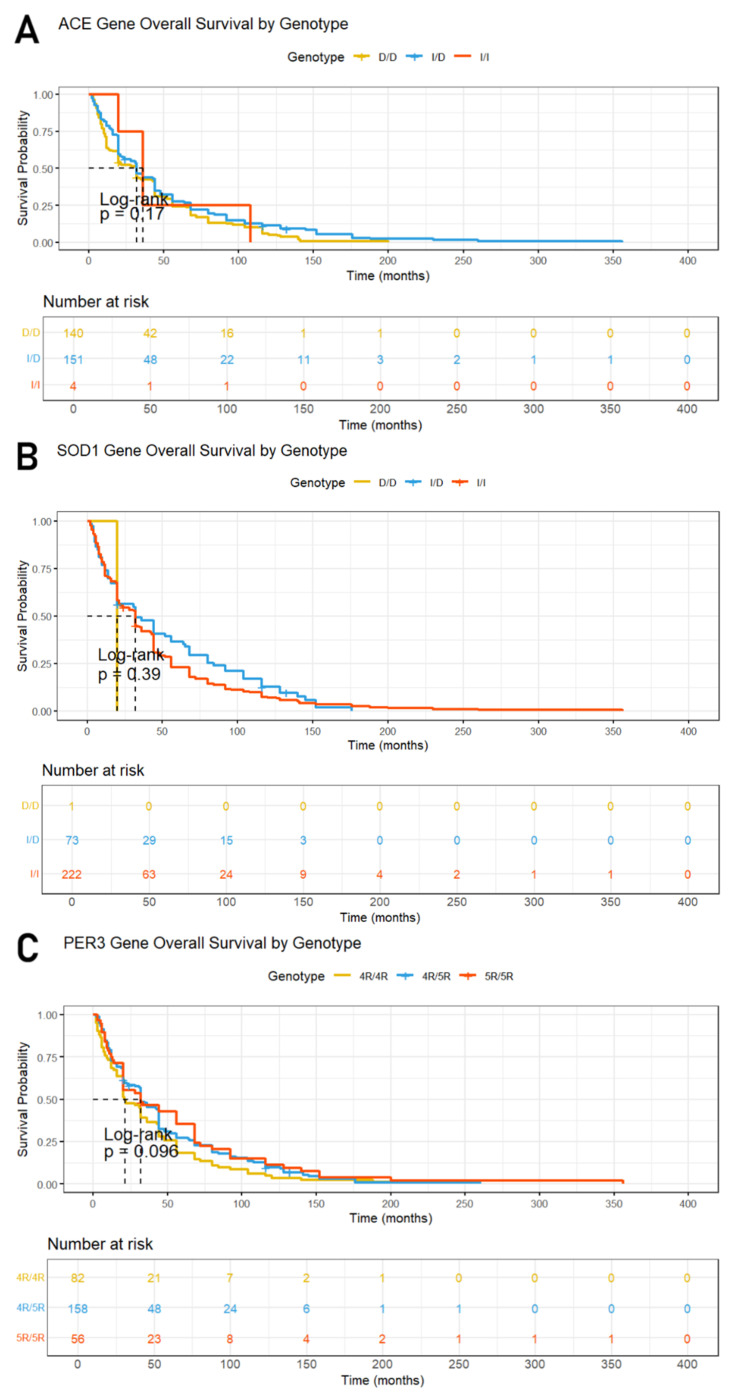
Kaplan-Meier curves and the log-rank test for overall survival (OS) according to (A) ACE (rs1799752), (B) PER3 (rs57875989) and (C) SOD1 (rs36232792) gene polymorphism genotypes.

Kaplan-Meier survival curves and the log-rank test were employed to evaluate overall survival rates among breast cancer patients, as presented in Figure 1. The median follow-up time for the study cohort was three years, during which five deaths occurred. The results showed no significant differences in survival times related to the *ACE* (rs1799752), *SOD1* (rs36232792) and *PER3* (rs57875989) variants, as evidenced by p-values of 0.17, 0.39 and 0.096, respectively, indicating that these genetic polymorphisms do not significantly impact patient survival in this study.

## Discussion

Breast cancer is the most common cancer type among women around the world and presents a major public health problem because of its diversity. Genetic predisposition represents a major risk factor for its development and progression. The onset and progression of several cancers have been linked to alterations in ACE expression, with elevated ACE levels often associated with more aggressive disease and poorer clinical outcomes. Furthermore, activation of the ACE/Ang-II/AT1R signaling pathway promotes cellular proliferation, invasion, angiogenesis, epithelial-mesenchymal transition and therapy resistance in cancer models 27, 28.

The rs1799752 insertion/deletion (I/D) polymorphism in the *ACE* gene has been widely investigated for its potential involvement in breast cancer development. Studies have indicated that the D allele of rs1799752 is associated with significantly higher circulating levels of the enzyme encoded by *ACE* compared with the I allele 29. This functional difference may influence angiotensin II production and consequently modulate pathways involved in tumor growth and metastasis. Based on this mechanism, Koh et al. proposed that women carrying the low-activity I allele may have reduced production of angiotensin II, which could potentially confer a lower susceptibility to breast cancer 30. Therefore, the *ACE* I/D polymorphism may represent a potential biomarker for evaluating breast cancer risk and clinical outcomes 22, 31.

Our study has identified a relationship between breast cancer susceptibility and the rs1799752 variant in the *ACE* gene, which is in accordance with previous findings in different populations, including Egyptian 22, North Indian 32, Turkish 33 and Dutch cohorts 34, as well as meta-analyses conducted in Asian and Caucasian populations 31. However, two other meta-analyses reported conflicting findings, indicating no significant association between the *ACE* I/D polymorphism and breast cancer risk 35, 36. Similarly, no significant relationship between the *ACE* I/D polymorphism and breast cancer susceptibility was observed in a Pakistani population 37.

Superoxide dismutase 1 (SOD1) is a key enzyme involved in the regulation of reactive oxygen species (ROS) in breast cancer cells. While SOD2 primarily regulates ROS within the mitochondria and is often downregulated due to reduced SIRT3 activity—resulting in elevated mitochondrial ROS levels—cancer cells may compensate by overexpressing SOD1 in the cytoplasm and mitochondrial intermembrane space 38. This compensatory mechanism helps limit excessive superoxide accumulation and maintain mitochondrial integrity. Inhibition of SOD1 has been shown to induce mitochondrial damage, highlighting its essential role in protecting cancer cells from oxidative stress and supporting their survival and proliferation 38.

Evidence suggests that SOD1 plays an important role in breast cancer development and tumor progression, independent of specific oncogenic drivers. Elevated SOD1 expression has been associated with poorer survival outcomes and increased metastatic potential. Although SOD1 appears to be essential for cancer cell growth, it is not required for normal cell proliferation 39. The rs36232792 polymorphism, characterized by a 50-bp insertion/deletion in the promoter region of the *SOD1* gene, may influence transcriptional regulation. Experimental in vitro studies have demonstrated that the 50-bp deletion reduces promoter activity and mRNA expression by eliminating two Sp1 transcription factor binding sites 40. Consequently, carriers of the deletion allele may exhibit reduced SOD1 expression and impaired detoxification of reactive oxygen species (ROS), which could compromise genomic stability and contribute to interindividual differences in cancer susceptibility 21, 41.

Our study demonstrated that the rs36232792 polymorphism in the *SOD1* gene is significantly associated with increased breast cancer risk. This finding is consistent with previous research in the Iranian population, which also reported no significant link between this variant and breast cancer susceptibility 42. In contrast, studies in the Mexican population observed a significant association 43. These discrepancies suggest that the impact of the rs36232792 polymorphism on breast cancer risk may be influenced by population-specific genetic backgrounds and environmental factors, emphasizing the importance of considering such differences in genetic association studies.

The Period Circadian Regulator 3 (*PER3*) gene have been implicated in breast cancer, with evidence suggesting that PER3 expression is reduced in tumor tissues. Lower PER3 levels have been associated with advanced tumor stage, higher metastasis risk and more aggressive cancer features, including ER negativity, higher histological grade and increased recurrence 14, 44. PER3 appears to inhibit cancer progression by suppressing the MEK/ERK signaling pathway, which is crucial for cell proliferation and survival, highlighting its potential as a prognostic biomarker and therapeutic target 44. Located on chromosome 1p36, a region frequently deleted in breast cancer, *PER3* has also attracted attention due to its role in circadian regulation. Disrupted circadian rhythms and altered sleep patterns are linked to increased breast cancer risk, suggesting that circadian-regulating genes like *PER3* may influence susceptibility 45. Circadian genes, including *PER3*, can affect hormone secretion, further connecting circadian disruption with tumorigenesis 14.

The *PER3* gene contains a variable number tandem repeat (VNTR) polymorphism (rs57875989) in exon 18, which consists of four or five copies of a 54-bp repeat sequence, which generates three genotypes: 4R/4R, 4R/5R, and 5R/5R. The 5-repeat allele introduces additional phosphorylation motifs within the PER3 protein, potentially altering phosphorylation-dependent regulation of circadian hormone secretion 46. These functional changes have been linked to differences in sleep patterns and disorders, and carriers of the 5/5 genotype have been reported to exhibit increased breast cancer susceptibility, particularly in premenopausal women 47,48.

Our study did not detect a significant association between the *PER3* rs57875989 polymorphism and breast cancer risk. This contrasts a previous study in Indian women, which reported an increased risk associated with the 5R genotype 46, while research in Caucasian women from Connecticut, USA, found that both heterozygous and homozygous carriers of the 5R allele exhibited elevated breast cancer risk, particularly among premenopausal individuals 49. In line with our findings, a study in the Dutch population, together with a comprehensive meta-analysis, reported only a non-significant trend toward increased breast cancer risk among carriers of the 5-repeat allele, most pronounced in homozygous individuals 24.

In our study, Hardy-Weinberg equilibrium (HWE) tests were performed for all selected polymorphisms in both cases and controls. Most polymorphisms conformed to HWE expectations, except for rs1799752 of the *ACE* gene, which showed a significant deviation (p < 0.05). This deviation may be influenced by several factors, notably the relatively small sample size, which can amplify the effects of genetic drift and affect expected genotype distributions.

HWE tests evaluate whether genotype frequencies in a population conform to expectations under random mating and no evolutionary pressures. Deviations can result from mutation, selection, gene flow or non-random mating. In the present case, the reason behind the deviation of the findings of the present study could be attributed to the presence of consanguineous marriages, which are common among the Jordanian population. Such non-random mating reduces genetic diversity and increases the likelihood of inheriting identical alleles, potentially distorting genotype frequencies and disrupting HWE 50, 51.

Previous research has indicated that deviation of certain SNPs from Hardy-Weinberg equilibrium does not necessarily reflect genotyping error and may instead reflect a true association with disease risk; consequently, excluding such variants may lead to the omission of biologically relevant genetic effects 52-55. Therefore, the basis of exclusion was not the violation of the Hardy-Weinberg equilibrium principle. The sensitivity analysis gave comparable results, validating the inclusion of this SNP in the accurate description of the population's genetic composition.

Statistical power analysis was conducted based on genotype frequencies under the most significant genetic models identified for each polymorphism. The *ACE* (rs1799752) variant showed significant associations with breast cancer susceptibility under both codominant and recessive models; however, the corresponding statistical power was 0.78, reflecting moderate power. Accordingly, these findings should be interpreted with caution and warrant validation in larger independent cohorts. These findings demonstrate the significance of incorporating power into the genotype-based inheritance model, as this would allow for a more accurate assessment of the genetic associations involved. However, the relatively low power exhibited by these findings indicates that additional research needs to be conducted on a larger scale in order to confirm these associations.

This study has several limitations that should be considered when interpreting the findings. Although the total sample size of 600 participants, including 300 cases and 300 controls, is comparable to many regional genetic association studies, it may still limit the statistical power to detect weak gene-disease associations. In particular, rare genotypes, such as the D/D genotype of the *SOD1* and the I/I genotype of *ACE*, were infrequent, which may increase confidence interval width and reduce the precision of odds ratio estimates. Therefore, the possibility of statistical bias or reduced sensitivity in detecting modest genetic effects cannot be excluded. Therefore, larger studies with greater sample sizes are warranted to validate the present findings and further clarify the role of these polymorphisms in disease susceptibility.

## Conclusion

The research provides essential insights into the relationship between *ACE*, *SOD1,* and *PER3* polymorphisms and breast cancer risk in Jordan. Our findings indicate that the rs1799752 polymorphism in the *ACE* gene is significantly associated with breast cancer susceptibility in Jordanian women, suggesting its potential as a genetic marker for risk assessment. Although these findings could enhance patient stratification and risk assessment, further large-scale and functional studies are needed to confirm these associations. This study is pioneering in its attention to Jordanian Arab descendants, investigating the impact of genetic polymorphisms and ethnicity on breast cancer risk. It contributes valuable insights into personalized medicine for breast cancer by suggesting the need to study understudied ethnic groups. The research emphasizes the importance of expanding scientific exploration to understand disease risk factors and mechanisms comprehensively.

## Supplementary Material

Supplementary table.

## Figures and Tables

**Table 1:** Genotyping parameters and PCR product characteristics.

Polymorphism	Genotyping	Annealing temperature	Allele	Fragment size (bp)
*ACE* (rs1799752)	Direct PCR	62	I D	490 190
*SOD1* (rs36232792)	Direct PCR	62	I D	297 247
*PER3* (rs57875989)	Direct PCR	68	5R 4R	257 193

**Table 2:** Hardy-Weinberg equilibrium values and minor allele frequencies of *ACE*, *SOD1* and *PER3* polymorphisms.

Gene	Control (n=300)			Cases (n = 300)		
	MA	MAF	HWE p-value	MA	MAF	HWE p-value
*ACE* (rs1799752)	I	30%	0.0053	I	27%	< 0.0001
*SOD1* (rs36232792)	D	15%	0.37	D	12%	0.062
*PER3* (rs57875989)	5R	46%	0.16	5R	46%	0.25

MA: minor allele. MAF: minor allele frequency. HWE: Hardy-Weinberg equilibrium

**Table 3:** Allele and genotype distributions with unadjusted and adjusted ORs for *ACE*, *SOD1*, and *PER3* variants.

Gene	SNP_ID	Allele/ Genotype	Control (n=300)	Cases (n=300)	Unadjusted OR (95% CI)	p-value	Adjusted OR (95% CI)	p-value
*ACE*	rs1799752	D I	422 (70%) 178 (30%)	438 (73%) 162 (27%)	1.00 1.14 (0.88-1.46)	0.337	---	---
		D/D I/D I/I	138 (46%) 146 (49%) 16 (5%)	142 (47%) 154 (51%) 4 (1%)	1.00 0.98 (0.70-1.36) 4.12 (1.34-12.62)	0.934 0.009	1.00 1.04 (0.72-1.51) 5.14 (1.38-19.03)	0.818 0.014
*SOD1*	rs36232792	I D	508 (85%) 92 (15%)	525 (88%) 75 (12%)	1.00 0.79 (0.57-1.10)	0.182	---	---
		I/I I/D D/D	217 (72%) 74 (25%) 9 (3%)	226 (75%) 73 (24%) 1 (0%)	1.00 1.06 (0.73-1.52) 9.37 (1.53-103.4)	0.849 0.011	1.00 0.92 (0.60-1.39) 7.21 (0.88-59.12)	0.685 0.066
*PER3*	rs57875989	4R 5R	325 (54%) 275 (46%)	324 (54%) 276 (46%)	1.00 0.99 (0.79-1.27)	0.999	---	---
		4R/4R 4R/5R 5R/5R	94 (31%) 137 (46%) 69 (23%)	82 (27%) 160 (53%) 58 (19%)	1.00 0.75 (0.52-1.09) 1.04 (0.65-1.66)	0.129 0.907	1.00 0.13 (0.02-1.06) 0.14 (0.02-1.14)	0.057 0.066

P-values < 0.0167 (0.05/# of variants, 0.05/3 = 0.0167 after applying multiple comparisons) are considered significant.

**Table 4:** Analyses of *ACE, SOD1*, and *PER3* polymorphisms using different genetic models.

Polymorphism	Model	Genotype	Cases (%)	Controls (%)	OR (95% CI)	P value
*ACE* (rs1799752)	Dominant	D/D I/D-I/I	142 (47.3%) 158 (52.7%)	138 (46%) 162 (54%)	1.00 1.06 (0.77-1.45)	0.74
	Recessive	D/D-I/D I/I	296 (98.7%) 4 (1.3%)	284 (94.7%) 16 (5.3%)	1.00 3.82 (1.33-10.97)	0.011
	Overdominant	D/D-I/I I/D	146 (48.7%) 154 (51.3%)	154 (51.3%) 146 (48.7%)	1.00 0.90 (0.65-1.24)	0.51
*SOD1* (rs36232792)	Dominant	I/I I/D-D/D	226 (75.3%) 74 (24.7%)	217 (72.3%) 83 (27.7%)	1.00 1.17 (0.81-1.68)	0.4
	Recessive	I/I-I/D D/D	299 (99.7%) 1 (0.3%)	291 (97%) 9 (3%)	1.00 6.51 (1.15-36.69)	0.021
	Overdominant	I/I-D/D I/D	227 (75.7%) 73 (24.3%)	226 (75.3%) 74 (24.7%)	1.00 1.02 (0.70-1.48)	0.92
*PER3* (rs57875989)	Dominant	4R/4R 4R/5R-5R/5R	82 (27.3%) 218 (72.7%)	94 (31.3%) 206 (68.7%)	1.00 0.82 (0.58-1.17)	0.28
	Recessive	4R/4R-4R/5R 5R/5R	242 (80.7%) 58 (19.3%)	231 (77%) 69 (23%)	1.00 1.25 (0.84-1.85)	0.27
	Overdominant	4R/4R-5R/5R 4R/5R	140 (46.7%) 160 (53.3%)	163 (54.3%) 137 (44.7%)	1.00 0.74 (0.53-1.01)	0.95

P-values < 0.0167 (0.05/# of variants, 0.05/3 = 0.0167 after applying multiple comparisons) are considered significant.

**Table 5:** Association between *ACE*, *SOD1*, and *PER3* polymorphisms and clinical features in breast cancer patients.

Clinical Outcome	ACE	SOD1	PER3
Age	0.374^b^ 0.6883	1.8458^b^ 0.160925	2.21639^b^ 0.110814
Age at BC Diagnosis	0.04081^b^ 0.96	1.40986^b^ 0.245812	3.36033^b^ 0.036067
Stage of BC	3.7051^b^ 0.05727	1.59214^b^ 0.208816	1.50217^b^ 0.227998
Age at First Pregnancy	0.03022^b^ 0.9702	2.95502^b^ 0.054207	0.60699^b^ 0.545978
Body Mass Index	2.0104^b^ 0.1358	2.21565^b^ 0.110895	1.1581^b^ 0.315562
Age of Menarche	0.9335^b^ 0.3945	2.88466^b^ 0.057598	0.82813^b^ 0.438019
Age of Menopause	0.08086^b^ 0.9223	1.5338^b^ 0.217803	2.72881^b^ 0.067402
Breastfeeding Status	2.0784^a^ 0.35373	0.755^a^ 0.6856	1.305^a^ 0.5207
Family History of Cancer	0.0492^b^ 0.97572	4.007^a^ 0.1349	1.016^a^ 0.6017
Other Types of Cancer	4.6375^a^ 0.098396	1.502^a^ 0.4719	2.877^a^ 0.2373
Polycystic ovary syndrome (PCOS)	0.355^a^ 0.8374	0.78^a^ 0.6771	0.9743^a^ 0.614369
Uterine Fibroid	0.42^a^ 0.8106	2.347^a^ 0.3093	0.129^a^ 0.9375
Benign Breast Tumor	0.086^a^ 0.9579	1.489^a^ 0.4750	2.4742^a^ 0.290227
Estrogen Receptor	0.101^a^ 0.9508	0.902^a^ 0.6370	1.317^a^ 0.5176
Progesterone Receptor	0.098^a^ 0.9522	0.396^a^ 0.8204	0.505^a^ 0.7769
Human Epidermal Growth Factor Receptor 2 (HER2)	3.285^a^ 0.1935	2.373^a^ 0.3053	0.465^a^ 0.7925
Axillary Lymph Node Metastasis	1.697^a^ 0.4281	0.626^a^ 0.7312	2.074^a^ 0.3545
Lymph vascular Invasion	0.23^a^ 0.6315	0.053^a^ 0.9738	0.062^a^ 0.9695
Distant Metastasis	0.145^a^ 0.9301	1.775^a^ 0.4117	1.911^a^ 0.3846
Allergy	0.96^a^ 0.6188	1.232^a^ 0.5401	1.9413^a^ o.378829
Smoking	3.173^a^ 0.2046	0.802^a^ 0.6697	0.113^a^ 0.9451

^A^ Parson's Chi-squared test was used to determine genotype-phenotype association (chi-square value). ^b^ Analysis of variance (ANOVA) test was used to determine genotype-phenotype association (f-ratio value). P-values < 0.0167 (0.05/# of variants, 0.05/3 = 0.0167 after applying multiple comparisons) are considered significant.

## Data Availability

The data that support the findings of this study are available from the corresponding author upon reasonable request.
