# Supplementary material for: Assessing the Association Between Genetic Variants in ACE, SOD1, and PER3 and their Role in Breast Cancer Risk among Jordanian Women
Source: J Cancer. 2026 Jun 10;17(6):1195–205. doi: 10.7150/jca.133243 (PMC13280626; doi:10.7150/jca.133243)
Supplement: Supplementary file 1 — Supplementary table. [file jcav17p1195s1.pdf]

Supplementary Table S1. Multivariable binary logistic regression analysis of genotypes, covariates and demographic features as predictors of breast cancer risk.

Polymorphism**	Covariate	Odd ratio	Confidence interval 95 %	P value*
ACE (rs1799752)	Age	0.992	0.976 – 1.008	0.324
	BMI	0.980	0.950 – 1.010	0.192
	Smoking	0.805	0.491 – 1.319	0.389
	Age of Menarche	0.984	0.871 – 1.111	0.792
	Family history	2.658	1.835 – 3.850	0.000*
	I/I	5.138	1.387 – 19.034	0.014*
	I/D	1.044	0.722 – 1.511	0.818
	D/D	Reference		
SOD1 (rs36232792)	Age	0.994	0.978 – 1.010	0.431
	BMI	0.982	0.952 – 1.013	0.244
	Smoking	0.879	0.540 – 1.429	0.603
	Age of Menarche	0.976	0.864 – 1.102	0.693
	Family history	2.559	1.770 – 3.698	0.000*
	D/D	7.216	0.881 – 59.123	0.066
	I/D	0.917	0.603 – 1.393	0.685
	I/I	Reference		
PER3 (rs57875989)	Age	0.994	0.978 – 1.010	0.431
	BMI	0.982	0.952 – 1.013	0.244
	Smoking	0.879	0.540 – 1.429	0.603
	Age of Menarche	0.976	0.864 – 1.102	0.693
	Family history	2.559	1.770 – 3.698	0.000*
	5R/5R	0.139	0.017 – 1.135	0.066
	4R/5R	0.127	0.015 – 1.062	0.057
	4R/4R	Reference		

* P-values < 0.0167 (0.05/# of variants, 0.05/3 = 0.0167 after applying multiple comparisons) are considered significant.

**The reference category is the control
